# Human Papillomavirus E6/E7-Specific siRNA Potentiates the Effect of Radiotherapy for Cervical Cancer *in Vitro* and *in Vivo*

**DOI:** 10.3390/ijms160612243

**Published:** 2015-05-29

**Authors:** Hun Soon Jung, Nirmal Rajasekaran, Sang Yong Song, Young Deug Kim, Sungyoul Hong, Hyuck Jae Choi, Young Seok Kim, Jong-Sun Choi, Yoon-La Choi, Young Kee Shin

**Affiliations:** 1 Research Institute of Pharmaceutical Science, Department of Pharmacy, College of Pharmacy, Seoul National University, Seoul 151-742, Korea; E-Mails: hunsoonjung@abionbio.com (H.S.J.); nirmalpharma@gmail.com (N.R.); sungyoul@snu.ac.kr (S.H.); 2 ABION Inc. R&D Center, 9th Floor, HanWha Biz Metro Bldg, 242 Digital-ro, Guro-gu, Seoul 152-733, Korea; E-Mail: todnos@abionbio.com; 3 Department of Pathology, Samsung Medical Center, Sungkyunkwan University School of Medicine, Seoul 135-710, Korea; E-Mails: yodasong@hanmail.net (S.Y.S.); yla.choi@samsung.com (Y.-L.C.); 4 Department of Radiology, Kangwon National University Hospital, Kangwon-do 200-722, Korea; E-Mail: choihjmd@gmail.com; 5 Department of Radiation Oncology, Asan Medical Center, College of Medicine, University of Ulsan, Seoul 138-736, Korea; E-Mail: ysk@amc.seoul.kr; 6 The Center for Anti-cancer CDx, N-Bio, Seoul National University, Seoul 151-742, Korea; E-Mail: jscmd@naver.com; 7 Tumor Microenvironment Global Core Research Center, Seoul National University, Seoul 151-742, Korea

**Keywords:** E6, E7, siRNA, Concurrent Chemoradiation therapy (CCRT), radiotherapy, radiosensitizer, cervical cancer

## Abstract

The functional inactivation of TP53 and Rb tumor suppressor proteins by the HPV-derived E6 and E7 oncoproteins is likely an important step in cervical carcinogenesis. We have previously shown siRNA technology to selectively silence both *E6*/*E7* oncogenes and demonstrated that the synthetic siRNAs could specifically block its expression in HPV-positive cervical cancer cells. Herein, we investigated the potentiality of E6/E7 siRNA candidates as radiosensitizers of radiotherapy for the human cervical carcinomas. HeLa and SiHa cells were transfected with HPV E6/E7 siRNA; the combined cytotoxic effect of E6/E7 siRNA and radiation was assessed by using the cell viability assay, flow cytometric analysis and the senescence-associated β-galactosidase (SA-β-Gal) assay. In addition, we also investigated the effect of combined therapy with irradiation and E6/E7 siRNA intravenous injection in an *in vivo* xenograft model. Combination therapy with siRNA and irradiation efficiently retarded tumor growth in established tumors of human cervical cancer cell xenografted mice. In addition, the chemically-modified HPV16 and 18 E6/E7 pooled siRNA in combination with irradiation strongly inhibited the growth of cervical cancer cells. Our results indicated that simultaneous inhibition of HPV *E6/E7* oncogene expression with radiotherapy can promote potent antitumor activity and radiosensitizing activity in human cervical carcinomas.

## 1. Introduction

Cervical cancer is the second most common cancer in women worldwide. Infection of the cervix with high-risk human papillomavirus (HPV), such as types 16 or 18, is a major cause of cervical cancer. The E5, E6 and E7 oncoproteins encoded by HPV play a critical role in cervical carcinoma [1,2,3]. Although most HPV-associated cervical carcinomas, unlike many other cancers, carry the wild-type *TP53* gene, the levels of TP53 protein in these carcinomas remain remarkably low, because the protein is constantly targeted for degradation by the E6 viral protein [4,5]. In addition, the E7 binds to the retinoblastoma (RB) family of tumor suppressor proteins and disrupts RB/E2F complexes, thereby driving cell division [6]. The functional inactivation of TP53 and RB tumor suppressor proteins by the HPV-derived E6 and E7 oncoproteins is likely an important step in cervical carcinogenesis. Thus, the E6 and E7 proteins may be suitable targets for treating cervical cancer. The HPV16 E5 is a hydrophobic protein observed in the endoplasmic reticulum, Golgi apparatus and nuclear membrane of infected cells. The E5 oncoprotein displays transforming activity and is believed to enhance the oncogenic effect of E6 and E7. However, its mechanistic role is not clear during cervical carcinogenesis [7,8].

Recently, RNA interference (RNAi) has been developed as a novel therapeutic strategy and is currently in early stage clinical trials [9]. Many investigators have developed RNAi targeting *E6* or *E6*/*E7*, which promote the accumulation of TP53 and/or hypo-phosphorylated RB (pRB). This eventually leads to the induction of apoptosis and/or senescence in cervical carcinoma cells [10,11,12,13,14,15,16]. Previously, we reported that small interfering RNA (siRNA) targeting *E6*/*E7* in combination with cisplatin (*cis*-diamminedichloroplatinum II; CDDP) exerts a synergistic effect through the restoration of TP53 and RB/E2F; moreover, it has a more potent therapeutic effect for cervical cancer than either of the agents alone have [17]. Thus far, we have validated the E6/E7 siRNA as a potential chemosensitizer for use in cervical cancer. Recent studies have shown that chemotherapy effectively enhances radiosensitivity in cervical cancer, and CDDP enhances the radiosensitivity of HPV16-positive SiHa cells [18]. Published reports have shown that concurrent chemoradiation therapy (CCRT) restored the function of TP53 at a molecular level. Arsenic trioxide (As2O3) [19] and ubenimex, a aminopeptidase N (APN)/CD13 inhibitor [20] are also known to enhance the radiosensitivity of human cervical carcinoma cells *in vitro* and *in vivo*. Randomized clinical trials for cervical cancer have shown that concomitant treatment with CDDP and radiotherapy (RT) is better than RT alone at improving prognosis [21,22,23]. Therefore, CCRT is recommended as the first-line therapy for patients with stage II B or a higher stage disease, and it is also recommended as an adjuvant therapy following radical hysterectomy for patients with locally-advanced cervical cancer [24,25]. This practice guideline by CCRT has improved survival rates in patients with cervical cancer. However, whether synergistic therapeutic effects can be achieved by combination therapy with *E6/E7* silencing and RT remains to be determined. In the present study, we assessed the synergistic therapeutic effects of combination therapy with E6/E7 silencing and RT in HPV-positive cervical cancer. Most importantly, selected E6/E7-specific siRNA candidates in combination with RT enhanced the anti-tumor effects in cervical carcinomas.

## 2. Results and Discussion

### 2.1. Effect of HPV18 E6/E7-Specific Lead siRNAs in Combination with Radiation on Cervical Cancer Cells

**Figure 1:**
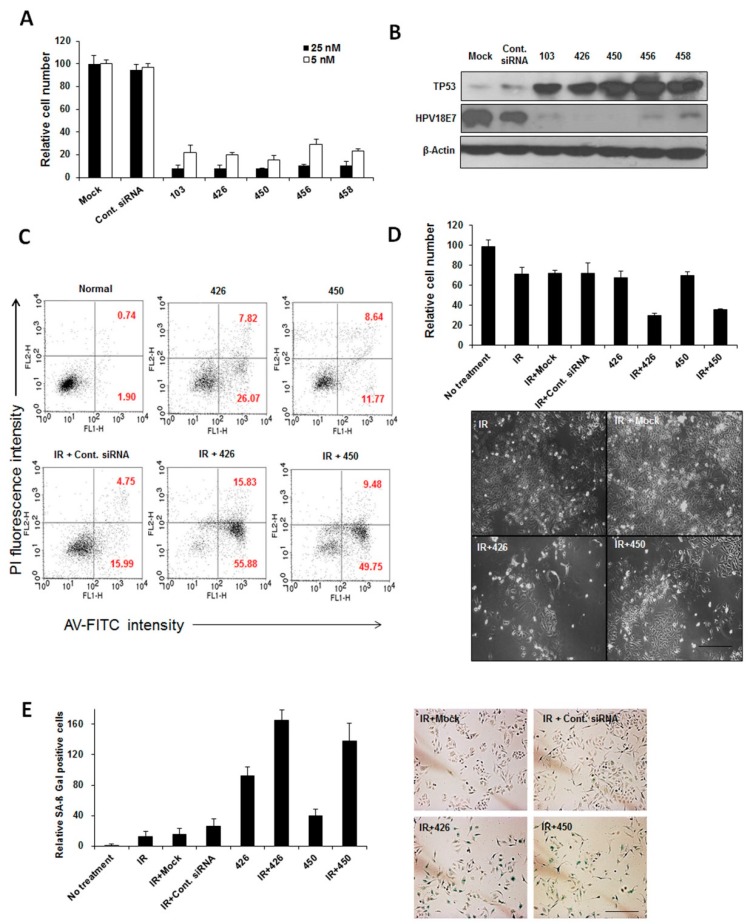
Screening and systematic analysis of HPV18 E6/E7-specific siRNA in combination with radiation. (**A**) Trypan blue assay showing the number of viable HeLa cells transfected with library siRNAs (103, 426, 450, 456 and 458). In these studies, HeLa cells were transfected with 5 or 25 nM of each siRNA. The number of cells was compared to reagent alone without siRNAs (mock); (**B**) Changes in TP53 and HPV18 E7 expression levels in HeLa cells following transfection with HPV18 E6/E7-specific library siRNAs were detected by Western blotting. β-actin was used as a loading control; (**C**) Annexin-V binding assay showing the percentage of apoptotic HeLa cells transfected with siRNA 426 or 450 for 28 h or siRNAs in combination with γ-irradiation; (**D**) The effects of E6/E7-specific siRNA 426 or 450 in combination with γ-irradiation on cell viability and morphology are shown. Scale bar: all are 200 μm; and (**E**) The effect of HPV E6/E7-specific siRNAs alone or combined with γ-irradiation (IR) on cellular senescence, Scale bar: all are 200 μm.

In a previous study, we revealed that E6/E7-specific siRNA, silencing both *E6* and *E7* mRNA, was more efficacious than E6-specific siRNA [17]. Moreover, the combination of E6/E7-specific siRNA and CDDP had a greater therapeutic efficacy in cervical cancer cells. The aim of this study was to identify siRNAs that have the potential to silence both HPV18- and 16-type *E6*/*E7* mRNA and simultaneously decrease E6/E7 protein-mediated degradation of TP53 in cervical cancer cells. A list of HPV18- and 16-type E6/E7 siRNA target sequences was generated (Tables S1 and S2). Ten library HPV-siRNAs were generated and screened for their silencing effects on HPV18, as well as HPV16-type *E6/E7*, respectively. The inhibition efficiency of siRNAs (103, 426, 450, 456 and 458) was shown by their significant anti-proliferative effect, unlike that of the other siRNAs (Figure S1a). To identify potential therapeutic siRNA, five out of 10 siRNAs were subjected to further analysis. As shown in Figure 1A, the inhibition of HeLa cell growth by at least 70% was achieved even at a low concentration. We also determined the cellular protein expression levels of E7, as well as TP53 in HeLa cells after *E6*/*E7* silencing by siRNAs. With regard to TP53 and E7 protein levels, we found that siRNA 426 or 450 was able to silence *E6*/*E7* expression more effectively than the other siRNAs (Figure 1B). Our results indicate that siRNA 426 and 450 showed a more robust effect than the other siRNAs did, in a dose-dependent manner (Figure S1b,c). After systematic screening of the library in triplicate, these results demonstrate that new, highly potent HPV18 siRNAs termed 426 and 450 are capable primary lead siRNAs. Similarly, on our screening analysis in SiHa cells (Figure S1c), HPV16-type-specific lead siRNAs termed 366 and 448 were selected along with siRNA 497 [16] for further studies.

Next, we have validated and compared the silencing effect of lead siRNAs in combination with radiation. We determined the silencing effect of HPV18 E6/E7-specific siRNA 426 or 450 in combination with radiation on apoptosis, cell viability and cellular senescence. HeLa cells were irradiated (2 Gy), transfected with siRNA 426 or 450, collected after a three-day exposure to the agents and analyzed by a flow cytometer. The percentages of Annexin-V-positive apoptotic cells are summarized in Figure 1C. siRNA 426 or 450, but not control siRNA, in combination with irradiation, significantly increased the percentage of Annexin-V-positive apoptotic HeLa cells. The apoptosis of cells transfected with siRNA 426 or 450 in combination with irradiation was significantly higher than that of the cells transfected with siRNAs alone. As shown in Figure 1D, growth of the cells receiving irradiation in the presence of siRNA 426 or 450 was significantly higher, and the cells showed morphological changes. Next, senescence-associated β-galactosidase (SA-β-Gal) activity was assessed three days after exposure to the combined-treatment of E6/E7-specific siRNAs with irradiation. Significantly higher SA-β-Gal activity was observed in the HeLa cells transfected with siRNA 426 or 450 in combination with irradiation, as observed by strong blue staining (Figure 1E). However, transfection of HeLa cells with E6/E7-specific siRNAs alone resulted in moderate SA-β-Gal activity. The ratio of SA-β-Gal-positive cells was summarized. Therefore, E6/E7-specific siRNA 426 or 450 in combination with radiation increased the cellular senescence of cervical cancer cells. Similar effects were observed with the HPV16-type-specific siRNA 497 (Figure S2). These results suggest that E6/E7-specific siRNAs augment the cytotoxicity of target tumor cells by radiation.

To determine the synergistic, additive or antagonistic effects of combination treatment of E6/E7-specific siRNAs with irradiation in HeLa cells, Chou–Talalay analysis was used. The synergistic activity of HPV18 E6/E7-specific siRNA 426 (5 or 25 nM) and HPV16 E6/E7-specific siRNA 497 (25 or 50 nM) with irradiation (2, 4 or 4.8 Gy) was demonstrated to be statistically significant (Table 1). Therefore, these findings confirm that E6/E7-specific siRNAs synergized with radiation to induce a therapeutic effect on cervical cancer cells.

**Table 1:** The synergistic therapeutic effect of siRNA 426 or siRNA 497 in combination with γ-irradiation was demonstrated by Chou–Talalay analysis.

426 siRNA (nM)	Radiation (Gy)	Cytotoxicity (%)	CI *
HPV18-Type HeLa Cells			
5	4.8	86	0.73
25	4.8	83	0.86
5	2	43	0.9
25	2	51	0.9
**497 siRNA (nM)**	**Radiation (Gy)**	**Cytotoxicity (%)**	CI *
**HPV16-Type SiHa Cells**			
50	2	78	0.63
25	2	68	0.71
50	4	77	0.80

* Combination index (CI) <1 indicates synergism.

### 2.2. In Vivo Effects of HPV18 E6/E7-Specific siRNA in Combination with Radiation

**Figure 2:**
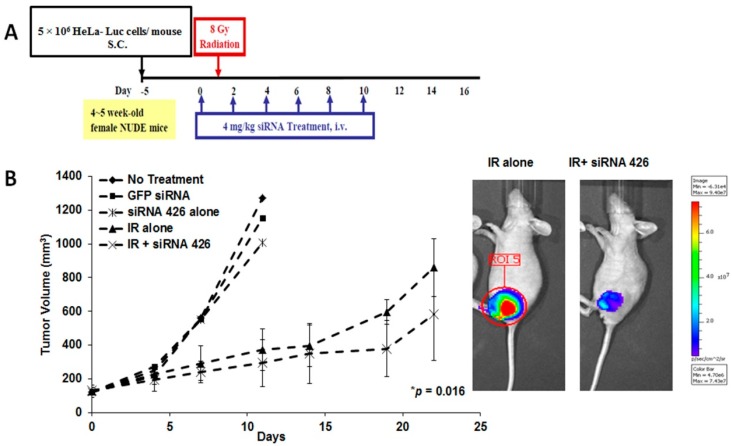

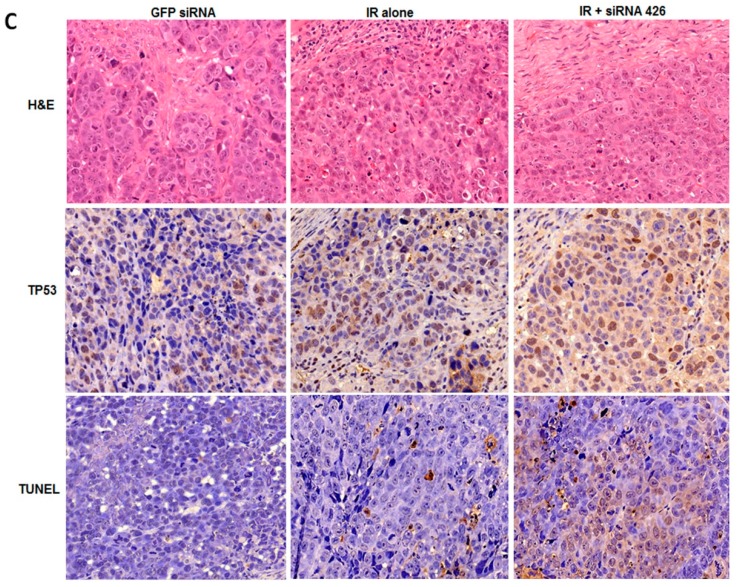
*In vivo* therapeutic effect of E6/E7-specific siRNAs in combination with radiation in human cervical cancer xenografted mice. (**A**) Treatment protocol; and (**B**) Luciferase activity was measured in live mice using the Luminescence Animal Imaging System, and tumor images were taken after 19 days. Untreated or control GFP-specific siRNA-treated mice served as controls. * *p* = 0.016 at Day 19, compared with γ-irradiation alone; and (**C**) H&E staining (×400), TP53 (×400) and TUNEL immunohistochemical (IHC) (×400) analysis on Day 25.

To examine the efficacy of HPV18 E6/E7-specific siRNA in combination with radiation therapy in the treatment of established human cervical cancer, luciferase-transfected HeLa cells were injected subcutaneously into the hind legs of nude mice (Figure 2A). Five days later, human tumor xenograft mice were injected intravenously with siRNA 426 six times at two-day intervals. On the day after the first siRNA 426 injection, the mice were irradiated with 8 Gy. As shown in Figure 2B, the mice receiving injections of siRNA 426 alone, control GFP-siRNA or untreated controls all developed tumors within 11 days after tumor challenge. However, three out of five mice receiving irradiation alone survived without tumor burden to the end of the experimental period (Day 21). The combination therapy of siRNA 426 with irradiation generated a superior therapeutic effect against established tumors compared with irradiation alone (*p* = 0.016). In the combination-treated group, all of the mice survived without tumor burden. Based on *in vivo* imaging, the combination therapy group, but not control groups, maintained low luciferase activity throughout the treatment period. These results show that HPV18 E6/E7-specific siRNA in combination with radiation significantly decreased tumor growth in a mouse xenograft model of human cervical cancer. For more dramatic *in vivo* efficacy, further studies are required to develop the siRNA nanoparticles that have specific targeted delivery systems [26,27,28,29].

Tumors treated with radiation therapy alone and combined with siRNA 426 were well demarcated and generally homogenous, with low cellular polymorphism. By contrast, tumors treated with green fluorescent protein (GFP) siRNA showed infiltrative margins, increased heterogeneity and high cellular polymorphism (Figure 2C). Consistent with these observations, TP53- and TUNEL-positive cells were markedly higher in the combination-treated mice than in the animals treated with GFP siRNA.

### 2.3. The Effect of Chemically-Modified E6/E7-Specific siRNAs on Silencing Efficiency and Serum Stability

Identification of chemically-modified potent siRNA is crucial for the therapeutic application of siRNA technology. Earlier reports have shown that 2ʹ-O-methyl (2ʹ-OMe) and 2ʹ-fluoro (2ʹ-F) modifications to siRNAs and Dicer-substrate RNAs can have significant effects on potency and serum stability without the loss of RNAi activity [30,31,32]. To increase the activity and stability of HPV E6/E7-specific siRNA, we designed and chemically modified (2ʹ-OMe, 2ʹ-F) the siRNAs (Table 2). Moreover, we examined the silencing efficiency and serum stability of the modified siRNAs.

**Figure 3:**
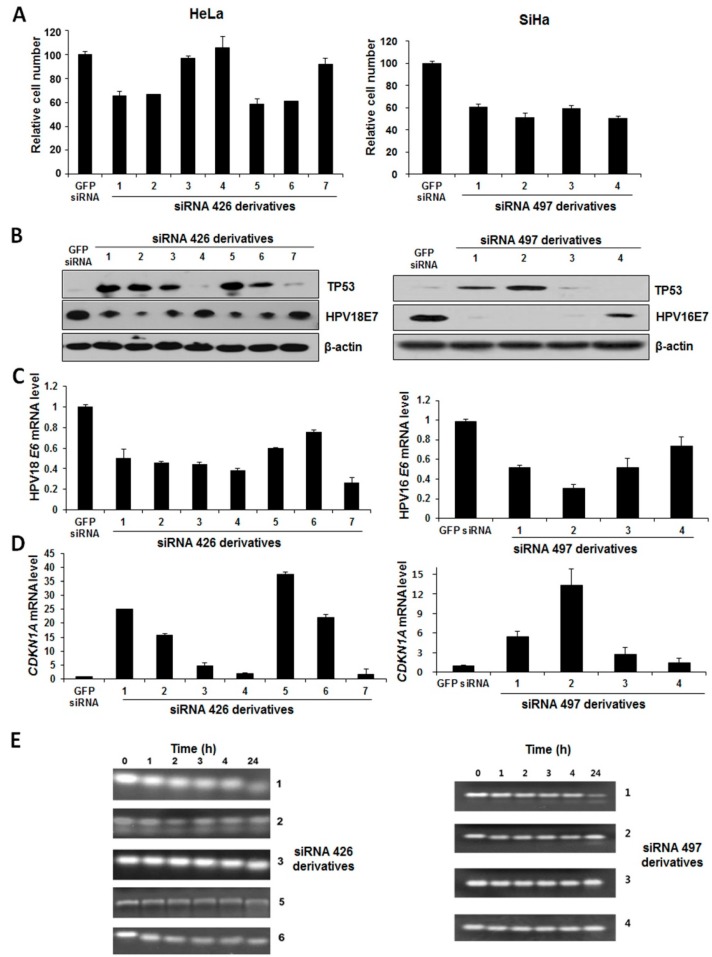
Determining the stability and silencing activities of chemically-modified derivatives of HPV16- and 18 E6/E7-specific lead siRNAs. (**A**) Trypan blue assay showing the number of viable HeLa cells transfected with 2ʹ-OMe-modified derivatives of siRNA 426 and SiHa cells transfected with 2ʹ-OMe modified derivatives of siRNA 497. GFP-specific siRNA (control siRNA) served as controls; (**B**) Silencing efficiency of 2ʹ-OMe-modified siRNA derivatives on E7 expression and changes in TP53 expression were also analyzed by Western blotting. β-actin was used as a loading control; (**C**) *E6* and (**D**) *CDKN1A* mRNA expression as determined by qRT-PCR; and (**E**) Gel electrophoresis analysis showing the serum stability of 2ʹ-OMe-modified siRNA derivatives. Unmodified (Lane 0) and modified siRNA 426 or 497 derivatives were analyzed by electrophoresis on 15% native polyacrylamide gels.

Based on our results, HPV18-type lead siRNAs (426 and 450) and HPV16-type lead siRNAs (366, 448 and 497) were chemically modified to enhance the nuclease stability. In our first line investigation, we examined the stability of siRNAs and the silencing efficiency by introducing 2ʹ-OMe and 2ʹ-F modifications. As shown in Figure 3A, the modified derivatives of siRNA 426 or 497 (Table 2) decreased the proliferation of HeLa or SiHa cells. In particular, cells receiving siRNA 426_d5 (derivative 5) and siRNA 497_d2 (derivative 2) showed significant cell growth inhibition compared to the other derivatives. We further assessed the efficiency of modified E6/E7-specific siRNAs by investigating the changes in TP53 and E7 protein levels. siRNA 426_d5 and siRNA 497_d2 were shown to affect the expression levels of TP53 and E7 (Figure 3B). Interestingly, their derivatives also significantly decreased *E6* mRNA levels (Figure 3C) and increased *CDKN1A* mRNA levels (Figure 3D). We next evaluated the serum stability of chemically-modified siRNAs by using gel electrophoresis analysis. The chemically-modified derivatives were comparatively more stable than the unmodified siRNAs (Figure 3E). We simultaneously determined the efficacy of other HPV16 E6/E7-specific modified siRNAs (366, 448) and HPV16 E6/E7-specific siRNA 450 (Supplementary Table S3). Among the modified derivatives, siRNA 450_d4 (derivative 4), siRNA 366_d2 (derivative 2) and siRNA 448_d2 (derivative 2) were selected as candidates based on the experimental results (Figures S3–S5). Similarly, in flow cytometric analysis, modified siRNAs transfection resulted in an increased percentage of apoptotic cells (Figure S6).

**Table 2:** List of selected siRNA derivatives used for chemical modification.

Name	Derivative Number		Sequence	Selected Candidates
**HPV18-type siRNA 426 (19mer)**	1	Sense (S)	5ʹ-caaccgagcacgacaggaa dTdT-3ʹ	
		Antisense (AS)	5ʹ-uuccugucgugcucgguug dTdT-3ʹ	
	2	6FC (S)	5ʹ- *c*aa*cc*gag*c*a*c*ga*c*aggaa dTdT-3ʹ	
		Antisense (AS)	5ʹ-uuccugucgugcucgguug dTdT-3ʹ	
	3	6FC (S)	5ʹ- *c*aa*cc*gag*c*a*c*ga*c*aggaa dTdT-3ʹ	
		4MeU (AS)	5ʹ-uucc ugucgugcucgguug dTdT-3ʹ	
	4	6FC (S)	5ʹ- *c*aa*cc*gag*c*a*c*ga*c*aggaa dTdT-3ʹ	
		6MeU (AS)	5ʹ- uuccugucgugcucgguug dTdT-3ʹ	
	5	5MeG (S)	5ʹ-caacc gagcacgacaggaa dTdT-3ʹ	d5
		Antisense (AS)	5ʹ-uuccugucgugcucgguug dTdT-3ʹ	
	6	5MeG (S)	5ʹ-caacc gagcacgacaggaa dTdT-3ʹ	
		4MeU (AS)	5ʹ-uucc ugucgugcucgguug dTdT-3ʹ	
	7	5MeG (S)	5ʹ-caacc gagcacgacaggaa dTdT-3ʹ	
		6MeU (AS)	5ʹ- uuccugucgugcucgguug dTdT-3ʹ	
**HPV16-type siRNA 497 (21mer)**	1	Sense (S)	5ʹ-gaccggucgauguaugucuug-3ʹ	
		Antisense (AS)	5ʹ-agacauacaucgaccggucca-3ʹ	
	2	Sense (S)	5ʹ-gaccggucgauguaugucuug-3ʹ	d2
		3MeU (AS)	5ʹ-agaca uacaucgaccggucca-3ʹ	
	3	6MeU (S)	5ʹ-gaccgg ucgauguaugucuug-3ʹ	
		Antisense (AS)	5ʹ-agacauacaucgaccggucca-3ʹ	
	4	6MeU (S)	5ʹ-gaccgg ucgauguaugucuug-3ʹ	
		3MeU (AS)	5ʹ-agaca uacaucgaccggucca-3ʹ	

Lower case letters represent ribonucleotides; Underlines are methyl modifications (2ʹ-OMe); Bold italics are F-fluoro modifications (2ʹ-F); S, sense strand; AS, antisense strand.

### 2.4. Synergistic Effect of E6/E7-Specific siRNA Pool in Combination with Radiation on Cervical Cancer Cells

In nature, the siRNAs generated by Dicer represent a pool of siRNAs that function together to silence gene expression. Pooled siRNAs generated in this manner are efficient silencers and have low off-target effects. We evaluated the apoptotic efficacy of HPV18-type siRNA pool (SP) using 10 nM of each siRNA and HPV16-type siRNA pool (SP) at 7 and 10 nM, because the preferred method to verify the identified siRNA candidates in screens by utilizing SP is to increase the efficiency of primary candidates. Interestingly, in both HeLa and SiHa cells, SP candidates could efficiently induce apoptosis even at a low concentration (Figure S7a).

**Figure 4:**
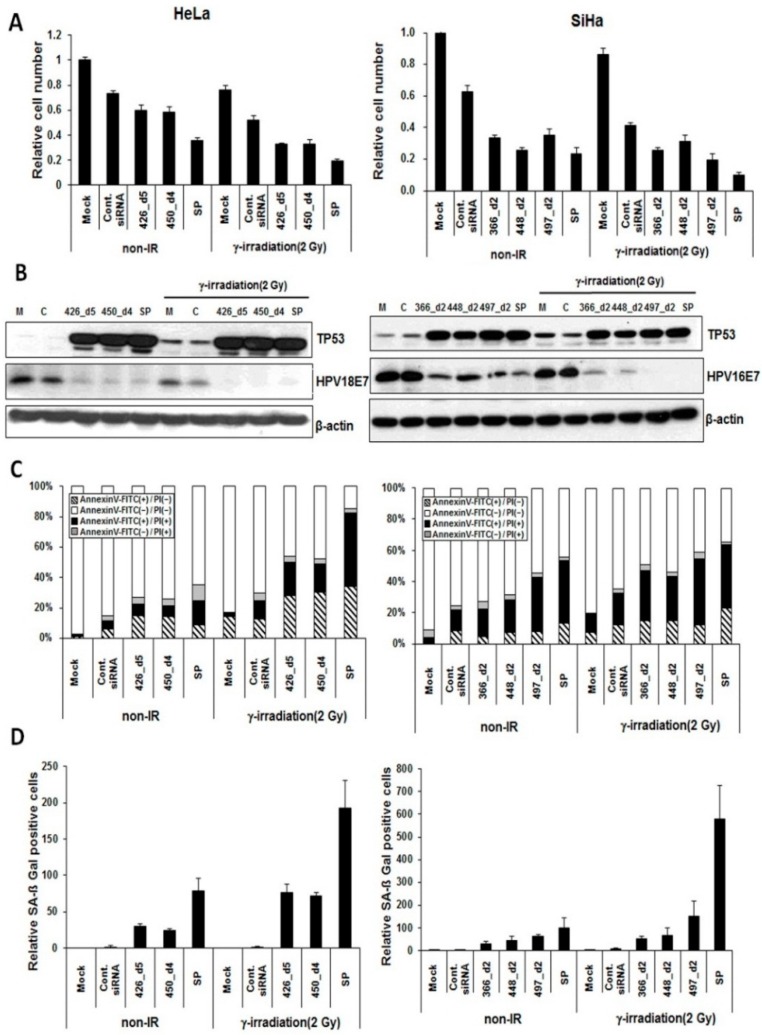
Synergistic effect of E6/E7-specific siRNA pools in combination with radiation on cervical cancer cells. HeLa cells were transfected with 20 nM of each selected siRNA derivative or siRNA pools (SP; 10 nM siRNA each) or in combination with γ-irradiation. Similarly, SiHa cells were transfected with 20 nM of each selected siRNA derivative or SP (7 nM siRNA each) or in combination with γ-irradiation. The number of cancer cells was determined by siRNAs alone (non-IR) or siRNAs in combination with γ-irradiation. Untransfected and control siRNA served as controls. (**A**) Trypan blue assay showing the number of viable cells transfected with siRNA pools, siRNA alone and in combination with γ-irradiation; (**B**) Western blotting analyses showing the expression of TP53 and HPV E7 proteins. M, mock; C, control siRNA; SP, siRNA pool; (**C**) Annexin-V and PI binding assay showing the percentages of apoptotic cells transfected with selected siRNA derivatives; and (**D**) The effect of HPV E6/E7-specific siRNAs on cellular senescence is also shown.

To assess whether the E6/E7-specific pooled siRNAs promote cell death and senescence, we first tested and compared the cytotoxicity of cervical cancer cells. Irradiated (2 Gy) HeLa or SiHa cells were transfected with pooled HPV E6/E7-specific siRNAs and analyzed after three-days’ exposure. SP-transfected HeLa or SiHa cells without irradiation served as the controls. As shown in Figure 4A, HPV18 E6/E7-specific SP (426_d5 and 450_d4) led to significantly higher growth inhibition than either siRNA 426_d5 or 450_d4 alone did. Pooled siRNAs in combination with irradiation significantly enhanced growth inhibition to levels greater than observed with other treatments. Similar results were observed in SiHa cells transfected with HPV16 E6/E7-specific SP (366_d2, 448_d2 and 497_d2).

We investigated the expression levels of TP53, HPV E7 (Figure 4B) and p16INK4a (Figure S7b). Irradiation with SP significantly induces the expression of TP53 and lowers the expression of p16INK4A and E7 than SP without irradiation and irradiation alone did. The *CDKN1A* mRNA level was increased substantially in SP-transfected cells with irradiation (Figure S7c). Furthermore, we observed and compared the effect of SP on apoptosis and cellular senescence in cervical cancer cells. SP alone and/or in combination with irradiation significantly increased the percentages of Annexin-V-positive apoptotic HeLa cells. The percentages of Annexin-V-positive apoptotic cell are summarized in Figure 4C. The degree of apoptosis in cells treated with pooled siRNAs in combination with irradiation was significantly higher than in cells treated with either siRNA 426_d5 or 450_d4 alone. In addition, siRNA 366_d2, 448_d2 and 497_d2 in combination with irradiation also increased the percentages of Annexin-V-positive cells in HPV16-positive SiHa cells. SA-β-Gal activity was assessed to determine the cellular senescence (Figure S7d), and the relative ratio of positive senescence cells is summarized in Figure 4d. The pooled siRNAs in combination with irradiation promoted higher cellular senescence in HeLa and SiHa cells. Moreover, the SA-β-Gal activity in pooled siRNA-transfected cells was significantly higher than in cells transfected with candidate siRNA alone. All of these results suggest that E6/E7-specific SP efficiently induced the cytotoxicity, apoptosis and cellular senescence of cervical cancer cells. Therefore, the low concentration of each siRNA in pooled siRNAs with radiation strongly inhibited the growth of cervical cancer cells.

Despite progress in the treatment of cervical cancer by using conventional chemotherapies and therapeutics in combination with concurrent radiation, this disease still remains fatal in the majority of patients. Thus, new therapeutic strategies are required to improve therapeutic options. The specific siRNAs targeting HPV-encoded oncogenes have definite therapeutic benefits in patients with cervical cancer. Earlier, we identified and validated some HPV18- and 16-type E6/E7-specific siRNA hits as chemosensitizers. Thus, we have established a proof-of-concept of combination therapy with E6/E7-specific siRNAs and CDDP [17].

According to the RNAi therapeutics pipeline, further studies were performed to discover highly potent E6/E7-specific lead siRNAs through siRNA library screenings. The anti-cancer effects may be more profound from those found in previous studies if more potent E6/E7-specific siRNAs are selected. In fact, newly identified siRNAs in this study elicited much stronger effects than E6/E7-specific siRNA hits, despite short-term exposure at low concentrations of siRNAs. Finally, five lead HPV18- and 16-type-specific siRNAs were selected, for which only siRNA 497 sequences were modified as an RNA-DNA chimera to enhance the target specificity in a previous study [33]. In addition, highly potent siRNAs, which were rationally designed, screened, validated for efficacy and chemically modified for stability, can mimic the pools generated in nature. These siRNA pools reduce the effective concentration and off-target effects of each siRNA, because each siRNA has a strong therapeutic effect. In this study, we have also used the pooled siRNAs to promote and accelerate the growth inhibition of cervical cancer cells. Our results confirmed that the pooled siRNAs in combination with radiation strongly inhibited the growth of cervical cancer cells. Therefore, the therapeutic benefit of highly potent siRNA pools means that they are of significant interest for drug development.

In the case of cervical cancer, CCRT is recommended as the first-line therapy. While this practice guideline has improved the survival rates, CCRT treatment fails in the majority of patients with residual cancers, adverse effects, local recurrences or distance recurrences common [21,22,23]. Therefore, there remain medically unmet needs for cervical cancer. First, in the case of early lesion recurrence and early lesions with high risk factors after surgery, such as margin or lymph node metastasis and microscopic parametrical involvement, as well as in advanced lesions beyond stage II B, it is important to maximize the effectiveness of CCRT and pelvic radiation in order to decrease recurrence and improve the survival rate. Next, there are also unmet needs for patients who have disease recurrence and metastasis after failure of CCRT. Clinical studies have been conducted to improve progression-free survival, overall survival and the reduction of severe adverse events [34,35,36,37]. Thus, to meet these medical needs, further studies are needed to evaluate combination therapy with CCRT and E6/E7-specific siRNAs.

## 3. Experimental Section

### 3.1. Cell Lines and Western Blotting

HeLa cells are an HPV18-positive human cervical cancer cell line. HeLa-Luc, a stably-transfected and bioluminescent human tumor cell line derived from HeLa cells, was obtained from Xenogen Corp. (Alameda, CA, USA). The HPV16-positive human cervical cancer cell line SiHa was obtained from the American Type Culture Collection (Manassas, VA, USA) with its identity verified by short tandem repeat analysis. The cells were routinely tested for mycoplasma. Primary antibodies against TP53, E7, p16INK4A and β-actin were used for Western blotting (for details, see Supplementary Materials and Methods).

### 3.2. In Vitro Models of siRNA Transfection and γ-Irradiation

siRNAs against HPV18 E6/E7 (426, 450), HPV16 E6/E7 (366, 448, 497) and control siRNA were synthesized from BIONEER (Dae-joen, Korea). Transfection was performed with siRNA using DharmaFect (Dharmacon, Lafayette, CO, USA), according to the manufacturer’s instruction. Chemically-modified (2ʹ-sugar modification by 2ʹ-OMe, 2-Fʹ) siRNA duplex derivatives were designed and synthesized by BIONEER (Dae-joen, Korea). All of the modified siRNA duplexes are complementary to the target sequences that are shown in Table 2. All siRNAs were suspended in DEPC-treated water to a final concentration of 5 μg/μL. For irradiation, the cells were seeded at a density of 5 × 10^5^ cells/100 mm dish. After 24 h, the cells were irradiated with 2–3 Gy of γ-irradiation in a GC 3000 Elan irradiator (MDS Nordion, Ottawa, ON, Canada). The following day, the cells were trypsinized and re-plated at a density of 3 × 10^5^ cell/100-mm dish.

### 3.3. Flow Cytometric Analysis and Senescence-Associated β-Galactosidase Assay

Apoptotic cells were stained with Annexin V-fluorescence isothiocyanate (FITC) and propidium iodide (BD PharMingen, San Diego, CA, USA), according to the manufacturer’s instructions, and were quantified by flow cytometry. For senescence analysis, the cells were fixed in 2% formaldehyde/0.2% glutaraldehyde and stained at pH 6.0 by using X-Gal (5-bromo-4-chloro-3-indolyl-β-d-galactopyranoside), by the method described previously [13]. SA-β-Gal-positive cells were counted in three representative fields.

### 3.4. Quantitative Real-Time PCR Analysis

Following single-agent or combination therapy, the expression of HPV18 *E6*, HPV16 *E6* and *CDKN1A* (p21^cip1^) was quantified using TaqMan quantitative real-time PCR (qRT-PCR) analysis (for details, see Supplementary Materials and Methods).

### 3.5. Serum Stability

siRNA duplexes (9 μg) were incubated at 37 °C in 90 μL 10% human serum. Aliquots of 12 μL were taken at various time points (0, 1, 2, 3, 4 and 24 h) and immediately frozen at −70 °C. RNAs were separated using 15% polyacrylamide-TBE under non-denaturing conditions and were photographed on a UV-transilluminator, after performing the EtBr staining [38].

### 3.6. In Vivo Xenografts, Imaging and Immunohistochemical Analysis

Balb/c-nude mice were challenged with 5 × 10^6^ luciferase-transfected HeLa cells into the hind legs. After five days, cationic liposome/siRNA (4 mg/kg body weight, 100 μL) was injected into the tail vein once every 48 h. The details of the cationic liposome preparation are in the Supplementary Materials and Methods. On the day after the first siRNA 426 injection, irradiation was given to the mice at 8 Gy. Tumors were visualized, and luminescence was measured 14–15 days post-implantation, using region-of-interest analysis and the *in vivo* imaging system (IVIS; Xenogen Corp., Alameda, CA, USA). This study was approved by the Institutional Review Board of Asan Medical Center (No. 2009-13-131).

Formalin-fixed and paraffin-embedded tumors were sectioned and stained with hematoxylin and eosin (H&E) or immunostained by using anti-TP53 antibodies. The terminal deoxynucleotidyl transferase (TdT)-mediated dUTP nick-end labeling (TUNEL) assay was performed to detect apoptosis (for details, see Supplementary Materials and Methods)

### 3.7. Chou and Talalay Analysis

To determine the pharmacological interaction between siRNA and irradiation, the Chou and Talalay analysis was used [24]. This method assesses synergy and antagonism by quantifying the divergence of the combination effect from the expected additive effect of the two therapeutic agents. A combination index (CI) was estimated from the dose-effect data. A CI of <1, =1 and of >1 indicates synergy, additive effects and antagonism, respectively.

## 4. Conclusions

We have confirmed the therapeutic synergy *in vitro* and *in vivo* between chemically-modified HPV18 or HPV16 E6/E7-specific siRNAs and radiation in cervical cancer. In addition, we confirmed the radiosensitizer effect of our HPV18 E6/E7-specific siRNA *in vivo.* Therefore, E6/E7-specific siRNA candidates have therapeutic potential as radiosensitizers in cervical cancer. Pooled HPV E6/E7-specific siRNAs in combination with radiation are more potent therapeutics than single siRNA to promote an anti-tumor effect in HPV-positive cervical cancer. Further work is warranted, encompassing the optimization of a suitable delivery system to improve cellular siRNA uptake for better therapeutic effect. To our knowledge, this is the first study showing the synergistic therapeutic effect of HPV E6/E7-specific siRNAs in combination with radiation for the treatment of cervical cancer. This information will be important for improving strategies involving siRNA therapeutics for treating patients with cervical cancer and possibly other tumors relating to HPV.

## Supplementary Materials

Supplementary materials can be found at http://mdpi.com/1422-0067/16/06/12243/s1.
