# Supplementary material for: Human Papillomavirus E6/E7-Specific siRNA Potentiates the Effect of Radiotherapy for Cervical Cancer in Vitro and in Vivo
Source: Int J Mol Sci. 2015 May 29;16(6):12243–60. doi: 10.3390/ijms160612243 (PMC4490442; doi:10.3390/ijms160612243)
Supplement: Supplementary file 1 [file ijms-16-12243-s001.pdf]

Supplementary Information

1. Western Blot

Whole-cell lysate were extracted using RIPA buffer (10 mM Tris (pH 7.4), 0.15 M NaCl, 5 mM EDTA, 1% Triton X-100, 0.5% deoxycholic acid sodium salt and 0.1% SDS). After centrifugation, the protein concentration of the supernatant was determined by BCA protein assay kit (Thermo Fisher Scientific Inc., Rockford, IL, USA). The protein samples were boiled in sample buffer for 5 min for complete denaturation and were resolved on a 6%, 10% or 15% polyacrylamide gel by SDS-PAGE and then transferred onto a 0.4 μm PVDF membrane (Millipore, Bilerica, MA, USA). After transfer, the blotted membrane was blocked with 5% nonfat dry milk in TBS-T (Tris-buffered saline with 0.1% Tween-20) and incubated with primary antibody at the appropriate final concentration followed by incubation with horseradish peroxidase-conjugated IgG (1:2000). Finally, western blot images were developed on photographic film using enhanced chemiluminescence (ECL) reagents. For each step, the membrane was washed with TBS-T three times for 10 min. The antibodies used for western blotting were as follows: anti-E7 (N-19), anti-p16INK4A (C-20), and anti-β-actin (C-2) were purchased from Sata Cruz Biotechnology, Santa Cruz, CA, USA, and TP53 (DO-7) was obtained from BD PharMingen, San Diego, CA, USA).

2. Detection of *E6* and *CDKN1A* ($p21^{cip1}$) Expression by qRT-PCR

Total RNA was extracted from the cells using a standard Trizol RNA isolation protocol (Invitrogen, Carlsbad, CA, USA) and then reverse-transcribed using oligo-dT primers and Powerscript reverse transcriptase (BD Biosciences, Palo Alto, CA, USA). Primers and probes for PCR were designed by TIB MOLBIOL (Berlin, Germany) and are shown in supplementary Table S4. qRT-PCR was performed using the LightCycler Real-time PCR Detection system (Roche Diagnostics, Basel, Switzerland). Cell and tissue mRNA levels were detected at 530 and 705 nm, respectively. The conditions for PCR were as follows: 95 °C for 10 min, 45 cycles at 95 °C for 10 s, 55 °C for 30 s, and finally 40 °C for 30 s. The $2(-\Delta\Delta C(t))$ method was used to calculate the relative expression. *E6* and *CDKN1A* ($p21^{cip1}$) expression was normalized to that of an internal control (β-actin).

3. Cationic Liposome Preparation

Cationic liposomes were kindly provided by Oh, Yu-Kyoung (Seoul National University, Seoul, Korea) and prepared using the conventional multilamellar vesicle technique.

1,2-Dioleoyl-sn-glycero-3-ethylphosphocholine (EDOPC), 3β-[*N*-(*N*′,*N*′-dimethylamino ethane)-carbamoyl] cholesterol and 1,2-diphytanoyl-sn-glycero-3-phosphoethanolamine were purchased from Avanti Polar Lipid, Inc. (Birmingham, AL, USA). Cationic lipid EDOPC and other lipids (2 μmole) were dissolved in chloroform. The lipids were mixed at appropriate weight ratios and the organic solvents were removed in a rotary evaporator under argon purge. The resulting thin films of lipids were hydrated with 1 mL of HEPES buffer (pH 7.4) and extruded three times though a 0.2 μm polycarbonate membrane filter using an extruder (Northern Lipids, Burnaby, BC, Canada). The complexation of EDOPC-based liposomes with siRNA was confirmed by a gel retardation assay on a 1.5% agarose gel. This cationic liposome/siRNA formulation shows less toxicity than commercial liposomal formulation (data not shown).

4. *In Vivo* Image Acquisition and Immunohistochemical Analysis

During image acquisition, mice were sedated by continuous inhalation of 1%–3% isoflurane. All the images were taken 15 min after the intraperitoneal injection of D-luciferin (150 mg/kg). Images and bioluminescent signals were acquired and quantified using the Living Image software (Xenogen Corp., Alameda, CA, USA). In addition, tumor size was measured using digital calipers, and tumor volumes were calculated as length × width × height × π/6 (mm^3).

Tumors were formalin-fixed, paraffin-embedded and sectioned at a thickness of 4 µm. All sections were then deparaffinized in xylene, rehydrated through serial dilutions of alcohol and washed in PBS (pH 7.2). PBS was used for all subsequent washes. For TP53 detection, sections were heated in a microwave oven twice for 5 min in citrate buffer (pH 6.0) and then incubated with a monoclonal anti-human TP53 (Santa Cruz Biotechnology, DO-1; at a dilution of 1:50) for 60 min at 25 °C and 30% humidity. For TP53 staining, the DakoCytomation CSA II Kit (Glostrup, Denmark) was used according to the manufacturer's instructions. Cells were counterstained with hematoxylin. Species-appropriate irrelevant isotype-matched monoclonal or polyclonal antibodies were used as negative controls. The terminal deoxynucleotidyl transferase (TdT)-mediated dUTP nick-end labeling (TUNEL) assay was performed to detect apoptotic cells using the ApopTag Peroxidase *In Situ* Apoptosis Detection Kit (Chemicon, Temecula, CA, USA) according to the manufacturer's instructions.

Table S1. List of designed HPV18 E6/E7 siRNA library target sequences.

siRNA ID No.	Nucleotide Position	Sequences
72	72–90	5′-ACTGCAAGACATAGAAATA-3′
97	97–115	5′-GTATATTGCAAGACAGTAT-3′
103	103–121	5′-GCAAGACAGTATTGGAACT-3′
113	113–131	5′-ATTGGAACTTACAGAGGTA-3′
426	426–444	5′-CAACCGAGCACGACAGGAA-3′
448	448–466	5′-CTCCAACGACGCAGAGAAA-3′
450	450–468	5′-CCAACGACGCAGAGAAACA-3′
456	456–474	5′-ACGCAGAGAAACACAAGTA-3′
458	458–476	5′-GCAGAGAAACACAAGTATA-3′
459	459–477	5′-CAGAGAAACACAAGTATAA-3′

The library of siRNA sequences targeted against HPV18 types *E6*/*E7* mRNA was created by using Dharmacon's siDESIGN website (http://www.dharmacon.com/).

Table S2. List of designed HPV16 E6/E7 siRNA library target sequences.

siRNA ID No.	Nucleotide Position	Sequences
39	39–57	5′-GGAGCGACCCAGAAAGTTA-3′
48	48–66	5′-CAGAAAGTTACCACAGTTA3′
68	68–86	5′-GCACAGAGCTGCAAACAAC-3′
78	78–96	5′-GCAAACAACTATACATGAT-3′
365	365–383	5′-AGCAAAGACATCTGGACAA-3′
366	366–384	5′-GCAAAGACATCTGGACAAA-3′
448	448–466	5′-TCAAGAACACGTAGAGAAA-3′
497	497–517	5′-GACCGGUCGAUGUAUGUCUUG-3′
573	573–593	5′-CACCUACAUUGCAUGAAUAUA-3′
752	752–772	5′-CUUCGGUUGUGCGUACAAAGC-3′

The library of siRNA sequences targeted against HPV16 types *E6*/*E7* mRNA was created by using Dharmacon's siDESIGN website (http://www.dharmacon.com/).

Table S3. List of HPV E6/E7 modified siRNA sequences.

Name	Derivative No.		Sequence	Selected Candidates
HPV16 Type siRNA 366 (19mer)	1	Sense (S)	5′-gcaaagacaucuggacaaa dTdT-3′	
		Antisense (AS)	5′-uuuguccagaugucuuugc dTdT-3′	
	2	3MeGU (S)	5′-gcaaagacaucuggacaaa dTdT-3′	d2
		Antisense (AS)	5′-uuuguccagaugucuuugc dTdT-3′	
	3	3MeGU (S)	5′-gcaaagacaucuggacaaa dTdT-3′	
		4MeG (AS)	5′-uuuguccagaugucuuugc dTdT-3′	
	4	3MeGU (S)	5′-gcaaagacaucuggacaaa dTdT-3′	
		4MeGU (AS)	5′-uuuguccagaugucuuugc dTdT-3′	
	5	4MeG (S)	5′-gcaaagacaucuggacaaa dTdT-3′	
		Antisense (AS)	5′-uuuguccagaugucuuugc dTdT-3′	
	6	4MeG (S)	5′-gcaaagacaucuggacaaa dTdT-3′	
		4MeG (AS)	5′-uuuguccagaugucuuugc dTdT-3′	
	7	4MeG (S)	5′-gcaaagacaucuggacaaa dTdT-3′	
		4MeGU (AS)	5′-uuuguccagaugucuuugc dTdT-3′	
HPV16 Type siRNA 488 (19mer)	1	Sense (S)	5′-ucaagaacacguagagaaa dTdT-3′	
		Antisense (AS)	5′-uuucucuacguguucuuga dTdT-3′	
	2	4MeG (S)	5′-ucaagaacacguagagaaa dTdT-3′	d2
		Antisense (AS)	5′-uuucucuacguguucuuga dTdT-3′	
	3	4MeG (S)	5′-ucaagaacacguagagaaa dTdT-3′	
		3MeG (AS)	5′-uuucucuacguguucuuga dTdT-3′	
	4	4MeG (S)	5′-ucaagaacacguagagaaa dTdT-3′	
		4MeU (AS)	5′-uuucucuacguguucuuga dTdT-3′	
HPV18 Type siRNA 450 (19mer)	1	Sense (S)	5′-ccaacgacgcagagaaaca dTdT-3′	
		Antisense (AS)	5′-uguuucucugcgucguugg dTdT-3′	
	2	Sense (S)	5′-ccaacgacgcagagaaaca dTdT-3′	
		4MeU (AS)	5′-uguuucucugcgucguugg dTdT-3′	
	3	4MeG (S)	5′-ccaacgacgcagagaaaca dTdT-3′	
		Antisense (AS)	5′-uguuucucugcgucguugg dTdT-3′	
	4	4MeG (S)	5′-ccaacgacgcagagaaaca dTdT-3′	d4
		4MeU (AS)	5′-uguuucucugcgucguugg dTdT-3′	

Lower case letters represent ribonucleotides; Underlined are Methyl modification (2′-OMe); (S) Sense strand; (AS) Antisense strand.

Table S4. List of qRT-PCR primers and probes.

Name	Sequence
HPV18 E6 forward	5′-GCG ACC CTA CAA GCT ACC TGA TC-3′
HPV18 E6 reverse	5′-ATG CAG CAT GGG GTA TAC TGT CTC-3′
TaqMan probe	5′-FAM-ACG GAA CTG AAC ACT TCA CTG CAA GAC A-TAMRA-3′
HPV16 E6 forward	5′-TGA CCT TCT ATG TCA CGA GCA-3′
HPV16 E6 reverse	5′-TGA TTA ACT CCA TCT ATT TCA TCG TT-3′
TaqMan probe	5′-FAM-CAG AGG AA- Dark quencher-3′
CDKN1A forward	5′-CGA AGT CAG TTC CTT GTG GAG-3′
CDKN1A reverse	5′-CAT GGG TTC TGA CGG ACA T-3′
TaqMan probe	5′-FAM-CAG AGG AG-Dark quencher-3′

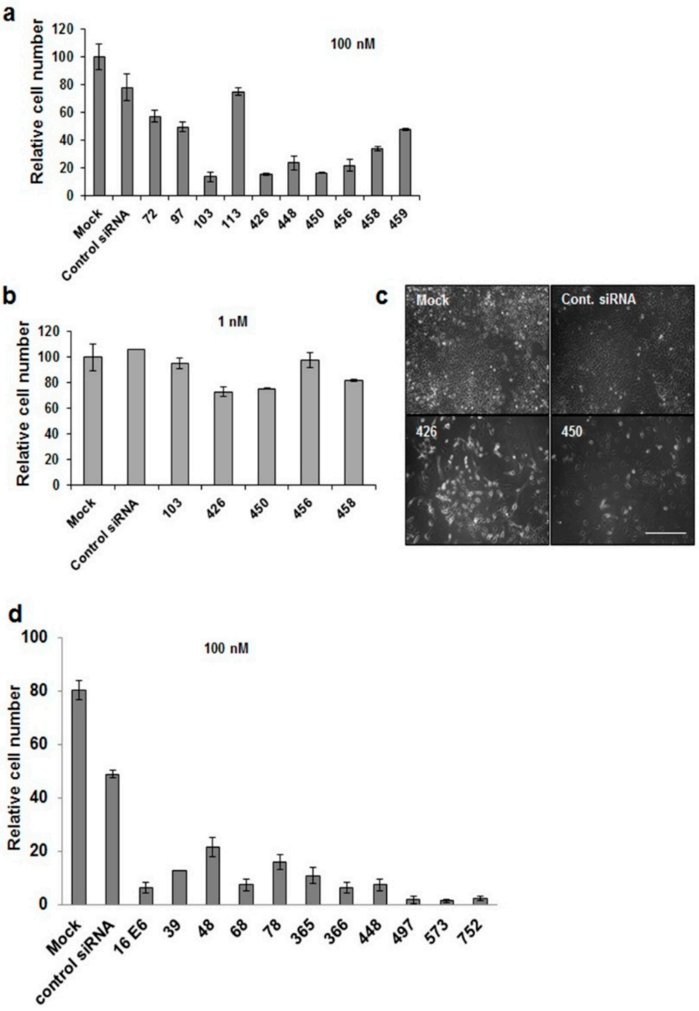

Figure S1. Screening analysis of HPV18 and 16 type E6/E7-specific library siRNAs. Trypan blue assay showing the number of viable HeLa cells transfected with HPV18 type library siRNAs. Summarized data for percentages of relative cell number are shown. In these studies, HeLa cells were transfected with 100 nM (**a**) or 1 nM (**b**) of each siRNA. The number of cells was compared to reagent alone without siRNAs (Mock); (**c**) Cell morphology changes are shown. Scale bar: all are 200 μm; and (**d**) similar screening analysis was performed by using HPV16 type library siRNA in SiHa cells. The error bars represent SD of three independent experiments.

Note 1: As shown in Figure 1c, growth of the cells receiving irradiation in the presence of siRNA 426 or 450 was significantly inhibited, and the cells showed morphological changes. The growth inhibition observed in combination therapy with siRNAs and irradiation was significantly higher than that in the cells transfected with siRNA 426 or 450 alone. HeLa cells with siRNA 426 or 450 alone failed to proliferate, and large adherent proliferating colonies formed after seven days (data not shown).

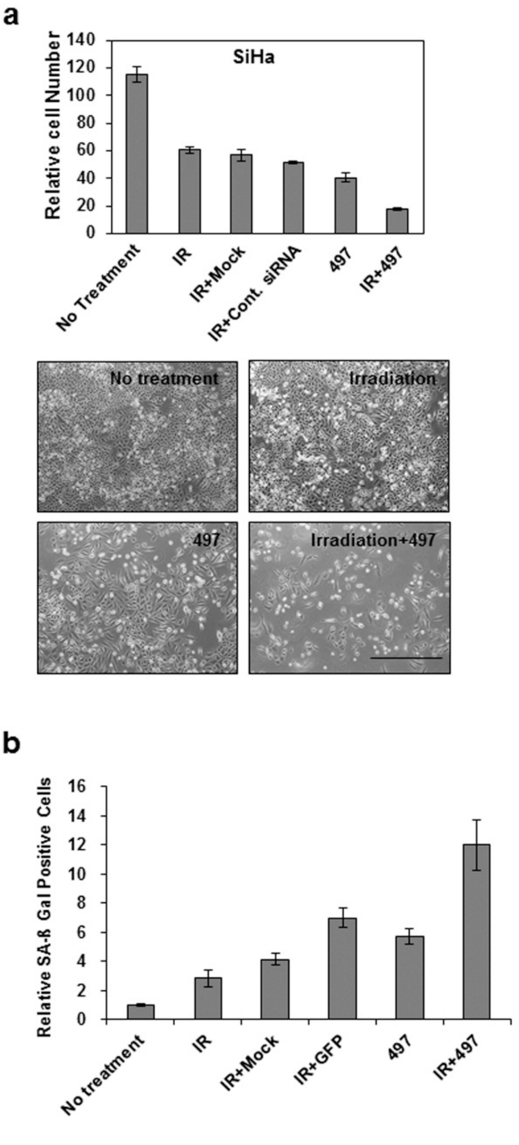

Figure S2. Effect of HPV16 type E6/E7-specific lead siRNA 497 in combination with radiation on apoptosis and cellular senescence. (**a**) Morphological changes in SiHa cells transfected with E6/E7-specific siRNA 497 in combination with ɣ-irradiation are shown. Summarized data for the number of viable cells are also shown. Scale bar: all are 200 µm; (**b**) SA-β-Gal positive cells were counted five days after transfection of siRNAs into SiHa cells. Summarized data for percentages of relative SA-β-Gal positive cells are shown. In these studies, SiHa cells were transfected with 20 nM siRNA. The error bars represent SD of three independent experiments.

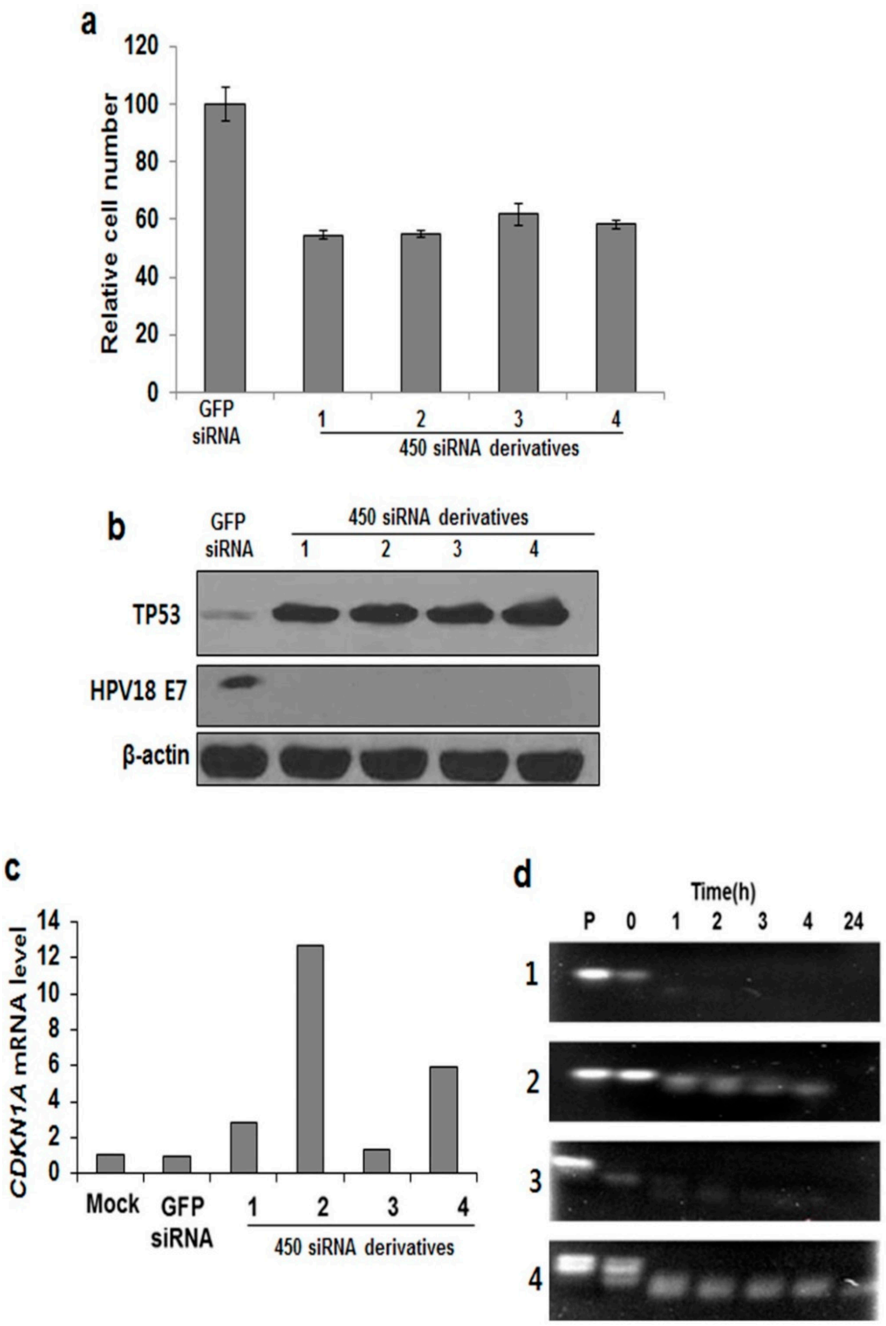

Figure S3. Determining the stability and silencing activities of chemically modified derivatives from HPV18 E6/E7-specific siRNA leads. (**a**) Trypan blue assay showing the number of viable HeLa cells after transfected with 20 nM of 2′-OMe modified derivatives of siRNA 450. GFP-specific siRNA served as controls; (**b**) Silencing efficiency of 2′-OMe modified siRNA derivatives on E7 expression and changes in TP53 expression was also analyzed by western blotting. β-actin was used as a loading control; (**c**) *E6* and *CDKN1A* mRNA expression as determined by qRT-PCR in HeLa cells. Expression was normalized to that of an internal control (β-actin); and (**d**) Gel electrophoresis analysis showing the serum stability of 2′-OMe modified siRNA derivatives. Unmodified (lane 1) and modified siRNA 450 derivatives were incubated in 10% human serum at 37 °C for the indicated times. Aliquots were analyzed by electrophoresis on 15% native polyacrylamide gels. The error bars represent SD of three independent experiments.

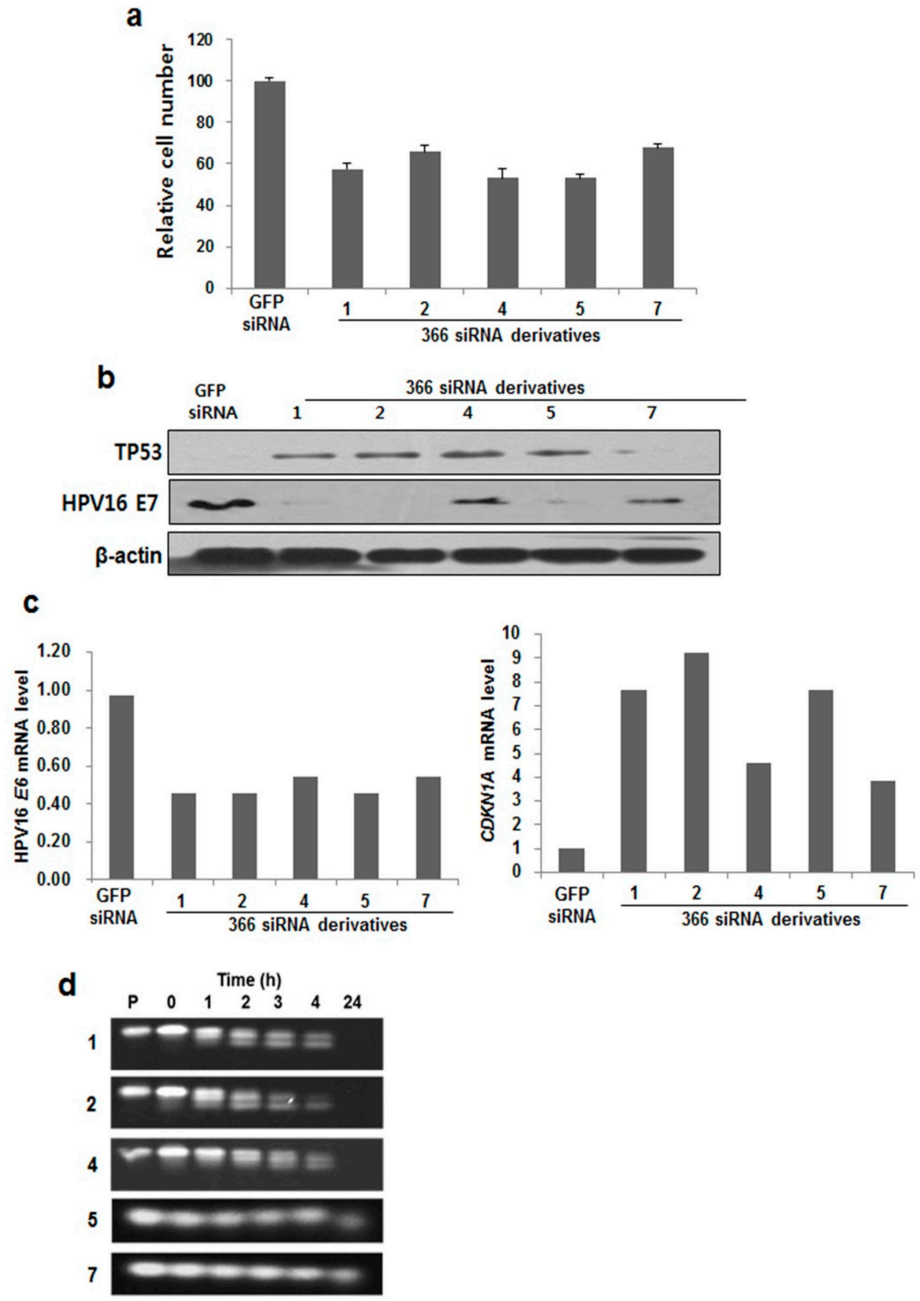

Figure S4. Determining the stability and silencing activities of chemically modified derivatives from HPV16 E6/E7-specific siRNA leads. (**a**) Trypan blue assay showing the number of viable SiHa cells after transfected with 20 nM of 2′-OMe modified derivatives of siRNAs 366. GFP-specific siRNA served as controls; (**b**) Silencing efficiency of 2′-OMe modified siRNA derivatives on E7 expression and changes in TP53 expression was also analyzed by western blotting. β-actin was used as a loading control; (**c**) *E6* and *CDKN1A* mRNA expression as determined by qRT-PCR in SiHa cells. Expression was normalized to that of an internal control (β-actin); and (**d**) Gel electrophoresis analysis showing the serum stability of 2′-OMe modified siRNA derivatives. Unmodified (lane 1) and modified siRNA 366 derivatives were incubated in 10% human serum at 37 °C for the indicated times. Aliquots were analyzed by electrophoresis on 15% native polyacrylamide gels. The error bars represent SD of three independent experiments.

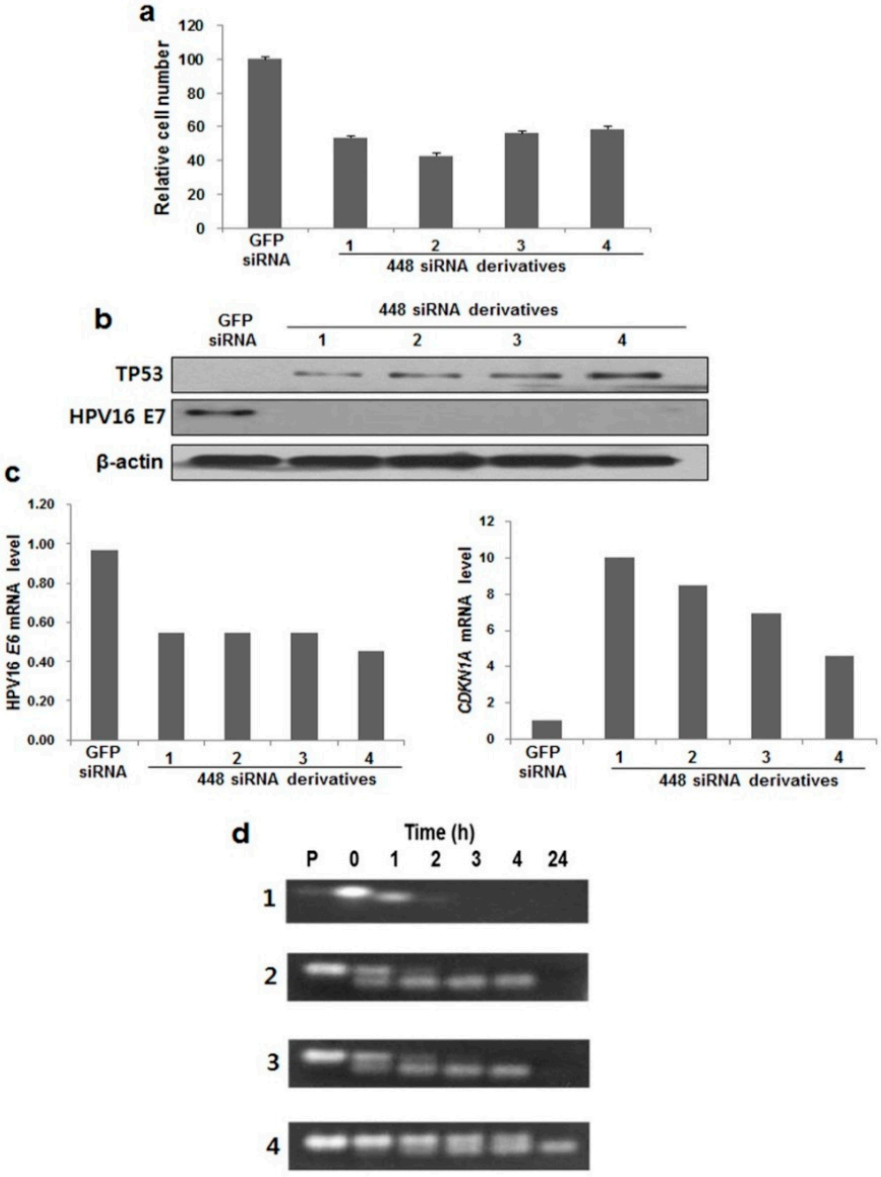

Figure S5. Determining the stability and silencing activities of chemically modified derivatives from HPV16 E6/E7-specific siRNA leads. (**a**) Trypan blue assay showing the number of viable SiHa cells after transfected with 20 nM of 2′-OMe modified derivatives of siRNAs 448. GFP-specific siRNA served as controls; (**b**) Silencing efficiency of 2′-OMe modified siRNA derivatives on E7 expression and changes in TP53 expression was also analyzed by western blotting. β-actin was used as a loading control; (**c**) *E6* and *CDKN1A* mRNA expression as determined by qRT-PCR in SiHa cells. Expression was normalized to that of an internal control (β-actin); and (**d**) Gel electrophoresis analysis showing the serum stability of 2′-OMe modified siRNA derivatives. Unmodified (lane 1) and modified siRNA 448 derivatives were incubated in 10% human serum at 37 °C for the indicated times. Aliquots were analyzed by electrophoresis on 15% native polyacrylamide gels. The error bars represent SD of three independent experiments.

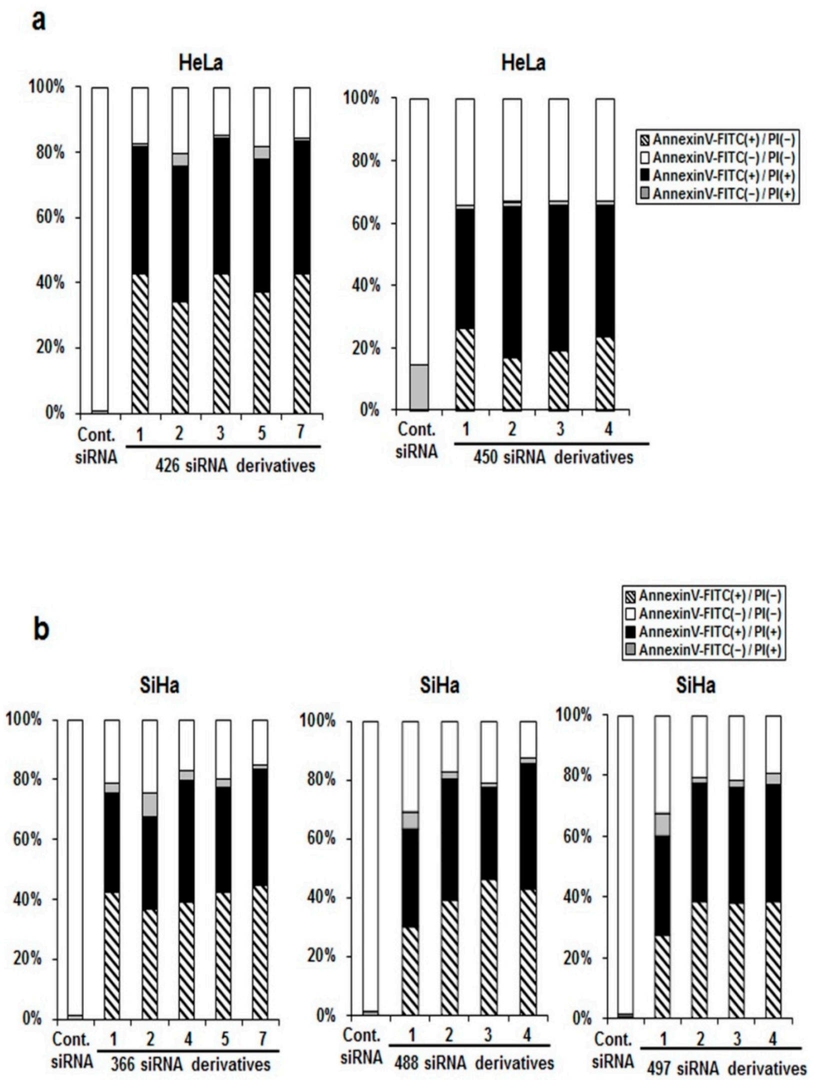

Figure S6. Apoptotic effect of chemically modified derivatives from HPV16 and 18 E6/E7-specific siRNA leads on cervical cancer cells (**a**) Flow cytometry analyses showing the percentages of apoptotic HeLa cells transfected with 10 nM of modified HPV18 E6/E7-specific siRNA 426 or 450; and (**b**) Showing the percentages of apoptotic SiHa cells transfected with 10 nM, 7 nM of modified HPV16 E6/E7-specific siRNA 366, 448 or 497. In these studies, apoptosis was significantly lower in HeLa or SiHa cells transfected with 20 nM of siRNA alone and control siRNA.

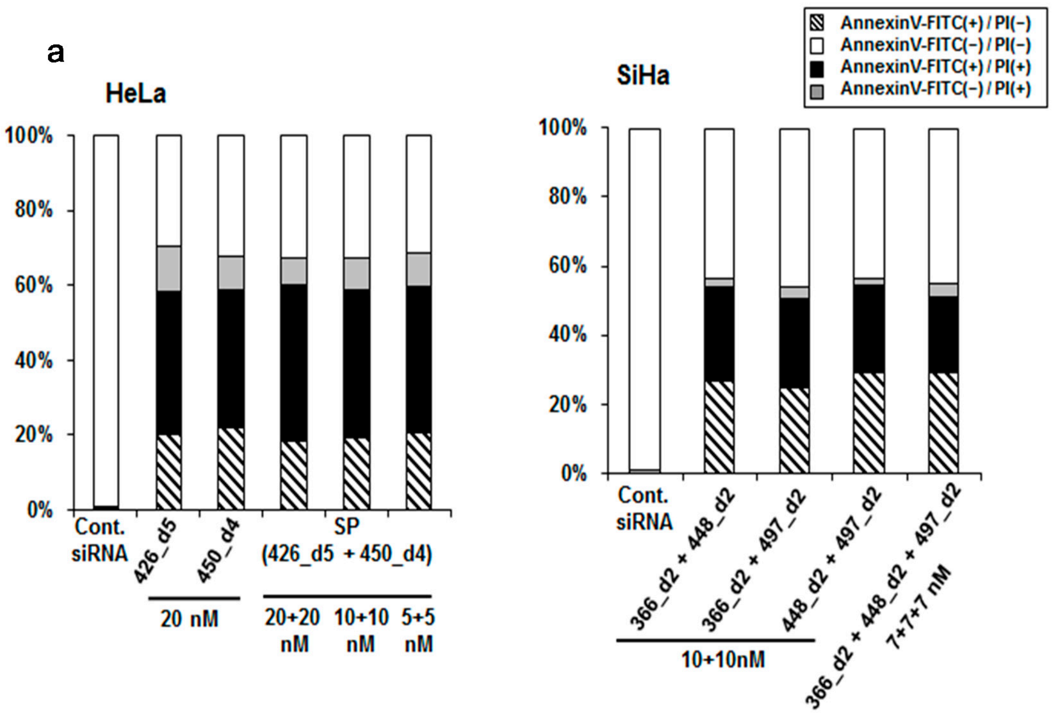

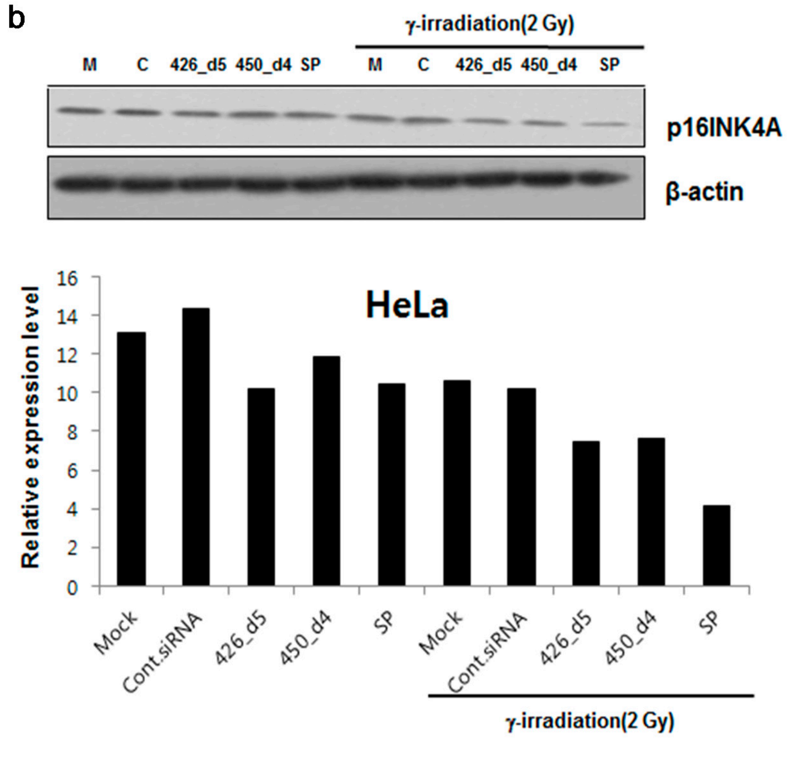

Figure S7. *Cont.*

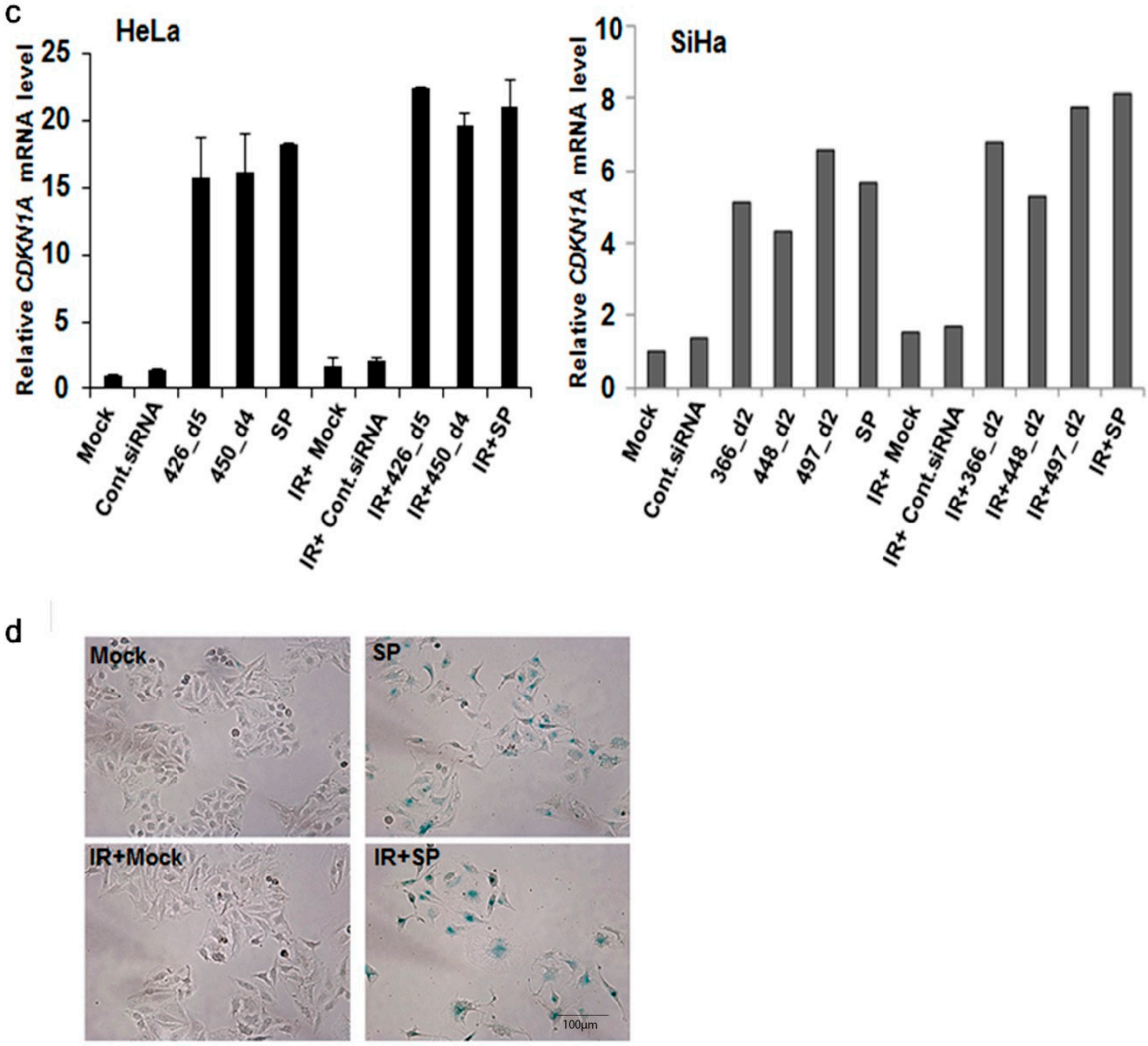

Figure S7. *In vitro a*nalysis of E6/E7-specific pooled siRNAs alone and/or in combination with radiation on cervical cancer cells. (**a**) Flow cytometry analyses showing the percentages of apoptotic HeLa and SiHa cells transfected with selected E6/E7 siRNA candidates either alone and/or in pooling; (**b**) HeLa cells were transfected with 20 nM of each siRNA or siRNA pools (SP; 10 nM siRNA each) in combination with irradiation. The relative expressions of p16INK4A protein expression levels are shown. Expression was normalized to that of an internal control (β-actin); (**c**) HeLa cells were transfected with 20 nM of each siRNA or siRNA pools (SP; 10 nM siRNA each) and SiHa cells transfected with 20 nM of each siRNA or siRNA pools (SP; 7 or 10 nM siRNA each) in combination with irradiation. The relative expression of *CDKN1A* mRNA levels is shown. Expression was normalized to that of an internal control (β-actin); and (**d**) SA-β-Gal positive cells after transfection with HPV16 and 18 type E6/E7-specific siRNA pools in combination with radiation on apoptosis and cellular senescence are shown; Scale bar: all are 100 µm.

Note 2: Molecular analysis of cellular changes in cervical intraepithelial neoplasia (CIN), identified an overexpression of the cyclin-dependent kinase inhibitor p16INK4a as a biomarker of an active

expression of the viral oncogene *E7* of all HR-HPV types [39–41]. However, it has been suggested that high expression level of p16INK4A in cervical cancer cells would not interfere with cell-cycle progression because of the presence of E7 oncoprotein, and that the upregulation of p16INK4A in cervical cancer is possibly the result of a feedback loop in the p16INK4A-Rb pathway [42,43].

All our results demonstrated that chemically modified HPV18 or HPV16 E6/E7- specific siRNAs in combination with radiation can simultaneously and effectively induce the down regulation both E6 and E7 expression, and consequently cells shows reduced proliferation and increased apoptosis, which may attribute to increased expression of TP53 as well as decreased the expression of cell cycle regulator p16INK4A, in HPV mediated cervical cancer cells. Based on previous studies which elaborated the role of HPV oncoproteins on p16INK4A expression was taken into consideration for our evaluation [44–46].
